# An Exploration of Student Perception Toward Interprofessional High-Fidelity Clinical Simulation

**DOI:** 10.1177/23821205241249594

**Published:** 2024-04-25

**Authors:** Michael Hannides, Rameez Tariq, Mark Holland, Polykarpos Angelos Nomikos, Rory McKelvin, Michelle Powell

**Affiliations:** 1 School of Clinical and Biomedical Sciences, 1796 University of Bolton, Bolton, UK

**Keywords:** Interprofessional education, high-fidelity simulation, physician associates, allied health professionals

## Abstract

**OBJECTIVES:**

Interprofessional education is recognized for its potential for collaboration and teamwork, reflecting clinical practice; however, existing literature for simulation-based interprofessional education does not include Physician Associate (PA) students. This initiative aimed to explore the students’ perception of interprofessional clinical simulation for PA students and allied health professional (AHP) students as part of our program development.

**METHODS:**

A high-fidelity simulation session was designed and conducted for volunteering students from the PA, paramedic science, and physiotherapy courses. We used a mixed-method electronic questionnaire consisting of 15 statements rated on a numerical rating scale (0-5) and four open-ended questions with unlimited free-text responses to explore student perceptions. Inductive thematic analysis was used for qualitative analysis. The session design was underpinned by Allport's (intergroup) contact hypothesis with an emphasis on mutual intergroup differentiation.

**RESULTS:**

Forty-six students participated in the simulation teaching, with 48% (n = 22) providing feedback. Overall student perception was mainly positive toward the interprofessional simulation; however, some barriers to learning were recognized. Based on the evaluation of our initiative and existing literature, we propose 5 top tips to promote an effective learning experience for students. (1) Understand the importance of interprofessional collaboration. (2) Establish clear roles. (3) Plan the scenarios in advance. (4) Maintain equal status between groups. (5) Provide clear instructions and expectations.

**CONCLUSION:**

To our knowledge, this is the first study of high-fidelity interprofessional simulation involving PA and AHP students. We successfully explored student perception which highlighted aspects that can impact learning. This pilot study demonstrated that interprofessional simulation is a feasible and acceptable form of learning for our students and highlighted how to improve future interprofessional simulation teaching sessions.

## Introduction

Allied health professionals (AHPs) constitute the third largest personnel within the National Health Service (NHS) and this is expected to rise following the commitment to workforce expansion in the NHS Long-Term Plan.^1^ Nevertheless, the number of applications to allied health courses has declined over the past 5 years.^2^ An independently commissioned report concluded this had, “*not delivered a step change in recruitment and student experience*,” and set out key recommendations, including collaboration through an interprofessional approach and the use of simulation to provide innovative methods of course delivery.^3^

Simulation has been widely used across numerous sectors with varying definitions.^4^ As a learning tool, simulation has the potential to bridge the educational gap from theory to practice in a safe and controlled learning environment.^5^ This is particularly beneficial in healthcare education, as it allows students to demonstrate competency in complex clinical situations without posing any significant risks to patients or colleagues.^6^ Health Education England (HEE) previously promised the development of a well-trained multiprofessional workforce through simulation-based education.^7^ The subsequent COVID-19 pandemic reinforced this commitment and also facilitated a revision in program standards from professional bodies including the Faculty of Physician Associates, increasing the permitted contribution of simulation toward total training hours.^8^

The guidelines or standards do not specify which type of simulation is permitted. Several levels of simulation have been proposed depending on the degree of complexity, technology, and experience which impact the overall effectiveness and veracity as an educational tool.^4^ High-fidelity simulation (HFS) is more realistic and can assess a broader range of knowledge and skills through performance by ascending Miller's pyramid of competency.^9^ High-fidelity simulation has several prerequisites, which can be more labor-intensive and costly, but well-suited for interprofessional training.^4^

Negative impressions of other professions can occur early in undergraduate training^10^; however, early interprofessional education may prevent these negative attitudes from developing.^11^ Similarly, poor collaboration and communication between disciplines has been associated with medical errors and adversely affect healthcare.^12,13^ Interprofessional simulation has already demonstrated the potential for improving teamwork^14^ and communication,^15^ although it is more frequently utilized in post-registration professional development in the workplace as opposed to students in a university setting.^16^

According to the Centre for the Advancement of Interprofessional Education, interprofessional education involves the integration of two or more professions to “learn from and about each other to improve collaboration and quality of care.”^17^ Existing literature for interprofessional education has included collaboration between various healthcare professions, including nursing, medical, occupational therapy, paramedic science, radiography, and pharmacy students.^14,15,18,19^ Unlike the well-established AHPs, Physician Associates (PAs) are a relatively new healthcare professional group, lacking a regulatory body and as such are dependent practitioners. While many AHPs have been represented in interprofessional simulation, no studies involving PAs exist to date.

Allied health professionals should be able to “work in partnership with other professionals,” “contribute effectively to work undertaken as part of a multidisciplinary team,” and “demonstrate effective and appropriate skills in communicating … to colleagues.”^20^ As a relatively new profession, trained in a different model, with overlapping expectations and competencies, it is unclear which position PAs will occupy relative to others. By evaluating the attitudes and experiences of the students, we aim to highlight benefits and barriers to learning which might potentially impact interprofessional relations in the future.

Within our School of Clinical and Biomedical Sciences at the University of Bolton, we have students across PA, paramedic science, and physiotherapy courses. This study aimed to explore the students’ perception of interprofessional high-fidelity clinical simulation.

## Methods

### Design

This study used a mixed-method approach to evaluate interprofessional simulation and explore student attitudes, experiences, and perceptions of the simulation. This study was part of an educational initiative from the University of Bolton School of Clinical and Biomedical Sciences. We designed and conducted a high-fidelity interprofessional simulation session to promote learning and collaboration between health professional students which ran from January 24 to 28, 2022.

Our questionnaire was designed by a group of experienced educators using evidence-based research^21^; however, it was not validated or piloted. Existing questionnaires for interprofessional collaboration were reviewed, however many were not validated and of those that were, some were for professionals rather than students and none existed specifically for PAs.^22^ Our questionnaire used a Likert-type scale consisting of 15 questions followed by 4 open-ended questions with unrestricted free-text responses. As well as assessing aspects of the fidelity of the simulation and self-perceived emotion, the questions were designed to include the principal factors incorporated into the “Readiness for Interprofessional Learning Scale” (RIPLS): teamwork and collaboration, professional identity, and roles and responsibilities.^23^ The RIPLS has been widely used in IPE for healthcare students and previously validated with a Cronbach alpha of 0.92.^24^

As this was a pragmatic pilot study to promote interprofessional simulation within the school of CBS, the developed questionnaire was felt to have content validity based on the consensus of the team however limitations will be considered in the discussion section.

### Participants

All students from the PA (MSc), paramedic science (BSc), and physiotherapy (BSc) courses were invited to attend the session voluntarily for this pilot session (n = 180). Prospective students were given participant information before signing a consent form in advance of the session. It was recognized the study might induce psychological stress or anxiety during the application for ethical approval. To mitigate potential negative emotions, students were informed in advance of the session, and the expectations and requirements were again outlined a week before the event. Students could opt out at any point.

No sample size calculation or analysis was conducted however we anticipated needing at least 10% (n = 18) to attend to ensure variability and adequate numbers for the simulation itself. Based on previous studies reviewed in the literature, this number as a minimum sample size could achieve thematic saturation.^25^

#### Inclusion Criteria

All current students from the paramedic science, PA, and physiotherapy programs at the University of Bolton were eligible to take part. Previous simulation experience was not a prerequisite.

#### Exclusion Criteria

Students who did not consent or attend the simulation event were excluded.

### Simulation Session

Background reading into preexisting literature and theoretical underpinning for our session design was essential. Simulation represents a form of experiential learning that allows the acquisition of applied knowledge^26^ and so the simulation was designed to challenge students, emulating real clinical practice but in a controlled environment. Allport's (intergroup) contact hypothesis demonstrates contact between groups can reduce prejudice and improve relationships provided four necessary conditions are met.^27^ Therefore, to promote collaboration, we designed the session to (1) maintain equal status between groups, (2) create common goals, (3) encourage cooperative working, and (4) provide institutional support.^27^

**Figure 1.**
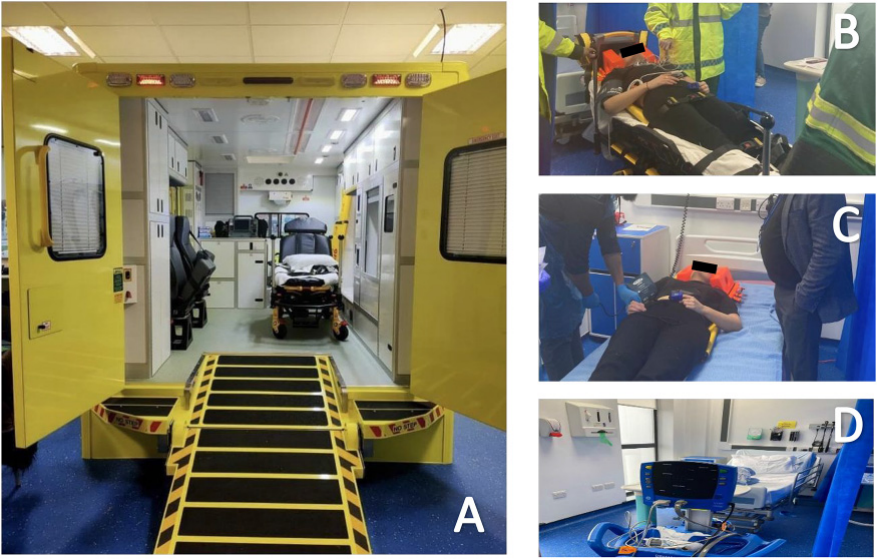
Photographic examples of the simulation facilities.

Expanding upon Allport's contact hypothesis, Hewston and Brown's emphasis on the group nature of contact influenced our session design. By keeping the scenarios relevant to each discipline, with frequent points of contact, we aimed to maintain distinctiveness to promote the salience of group boundaries and “mutual intergroup differentiation.”^28^ Clinical case scenarios were agreed upon and developed in advance by educators from the three participating professions. These scenarios were relevant and problem-centered, aligning with the principles of andragogy.^29^ For example, some case scenarios were tailored toward the prehospital setting utilizing our state-of-the-art simulation ambulance, or “*simulance*” (see Figure 1). Patients were then transferred to one of our simulation suites replicating an Emergency Department, where they would be assessed, stabilized, and treated (see Figure 1B and 1C). Patients were then discharged, relocated, or admitted to one of our longer-stay simulation wards for ongoing intervention (Figure 1D).

Our facilities and scenario design allowed for a high-fidelity and realistic simulation by reflecting the patient's journey across various settings. The scenarios were immersive and student-led, which allowed for an interprofessional approach to education with students from the paramedic science, PA, and physiotherapy programs in line with their curriculum and competencies. All staff were trained educators with various experiences in both clinical and educational practice and had a previous professional relationship with the students. These facilitators were briefed on their role, specifically to adopt a “laissez-faire” style of minimal intervention to encourage students to think, discuss, and problem-solve between themselves and others.^30^ To encourage involvement, additional scenarios relevant to each profession ran parallel as well as the use of manakins and equipment to practice clinical skills and establish clinical competency by allowing the ascension of Miller's pyramid through a practical demonstration of learning (shows/does).^9^

### Ethics

Ethical approval was obtained from the Bolton Research Ethics Committee (REC 20).

### Data Collection and Statistical Analysis

Students were invited to complete a post-session feedback questionnaire via an online survey (Supplementary file 1) within 1 week of their completed session. The survey required the student to declare their program of study but was otherwise anonymous. The Jisc online survey tool is designed for academic research and is both GDPR compliant and ISO 27001 standard certified. Feedback was collected and collated onto a single PDF document, which could not be edited, one week after the simulation event. Data were subsequently transcribed onto a Microsoft Access master database, which was stored in a secure, password-protected file, in keeping with university data protection regulations.

#### Quantitative Data

Students were initially asked to rate 15 statements on a numerical rating scale of 0 to 5, where 0 was the most negative response and 5 was the most positive. Students were then asked four open-ended questions with unlimited free-text responses to explore perceptions in more detail.

#### Qualitative Data

Inductive thematic analysis was used as the analytical method for the qualitative data.^31^ Open-text responses were transcribed, and these transcriptions were read several times by two independent authors (MHa and MP) to gain familiarity with the data. Although no interviews or focus groups were conducted in this study, a consolidated checklist was used.^32^

Author MHa was a module lead at the university and had completed his PG certificate in Medical Education and was working toward his Diploma. Author MP was head of the School of Biomedical and Clinical Sciences at the university and she had experience with research as part of her PhD. Both authors had experience with research and data analysis.

After coding the transcripts, an analytical framework was developed. The framework was applied to the rest of the transcripts, and a final coding framework was developed. The assessors then collaborated, and any disagreement was adjudicated by a third assessor (RT). An iterative process was used, and themes were developed.

## Results

### Quantitative Data

Forty-six students participated in the interprofessional simulation event and 22 (48%) completed a feedback form. Of the 22 responses, one was missing data on a single question and 6 forms (27%) had blank free-text responses.

**Figure 2.**
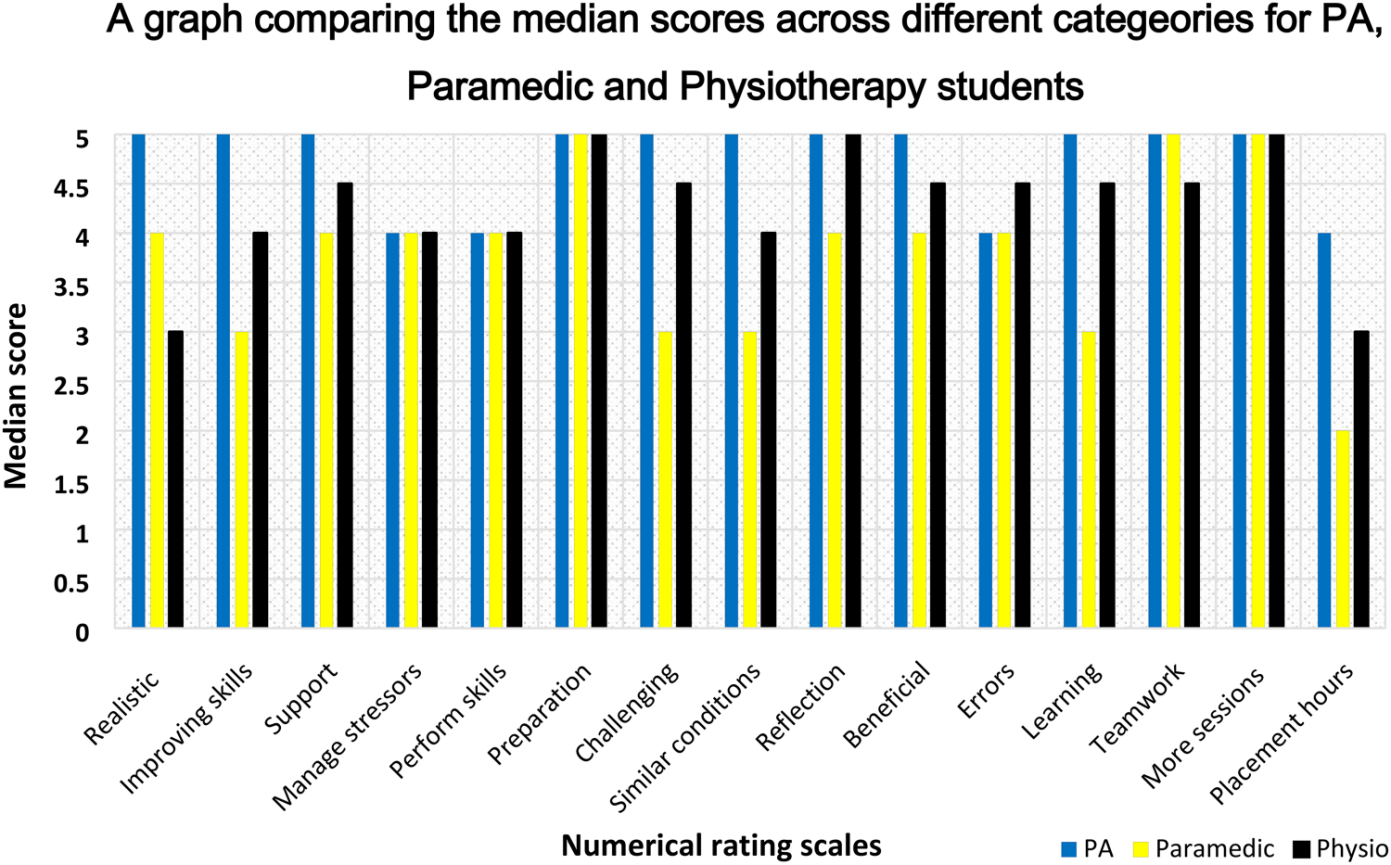
A graph comparing the median scores of Physician Associate (PA), paramedic and physiotherapy students across various categories.

The respondents included PA students (n = 11), paramedic science students (n = 9), and physiotherapy students (n = 2). The descriptive numerical statistics for all participants are summarized in Table 1. There was sufficient data to make a meaningful comparison between PA and paramedic scores (Figure 2).

**Table 1.** A Summary of Descriptive Statistics of Central Tendency and Variability.

**Statement**	**N**	**Min**	**Max**	**Mean**	**Median**
***The simulated event represented a realistic work environment***	22	1	5	3.7	4
***I am confident that I am improving my skills used in this scenario***	22	1	5	3.8	4
***I am confident that the simulation environment supports me in developing required skills***	22	1	5	4.2	5
***I am confident that I can manage the external stressors from the simulation***	22	1	5	3.8	4
***I am confident that I would perform the skills practiced, if required***	22	2	5	4.0	4
***I am confident repeated sessions…would be beneficial for preparation as a registered practitioner***	22	3	5	4.5	5
***I found the simulated event was challenging***	22	0	5	3.5	4
***I am confident that I was stressed to similar conditions…working in a real work environment***	22	1	5	3.6	4
***I am confident I can reflect on any areas for improvement and learn from them***	22	3	5	4.5	5
***I found this…beneficial to learning for my own practice and preparing me for real patients***	22	1	5	4.0	5
***Although errors may have occurred, I feel I will be more confident in my own practice***	22	1	5	4.0	4
***I feel that I have learnt more about working as a team with other professionals***	21	2	5	3.9	4
***I feel there is benefit to practicing and training with other professionals***	22	3	5	4.5	5
***I would want to do simulated events like this more frequently***	22	2	5	4.5	5
***I would be happy to have a percentage of placement hours to be replaced with this…type of simulated learning***	22	0	5	3.1	3

### Qualitative Results

**Figure 3.**
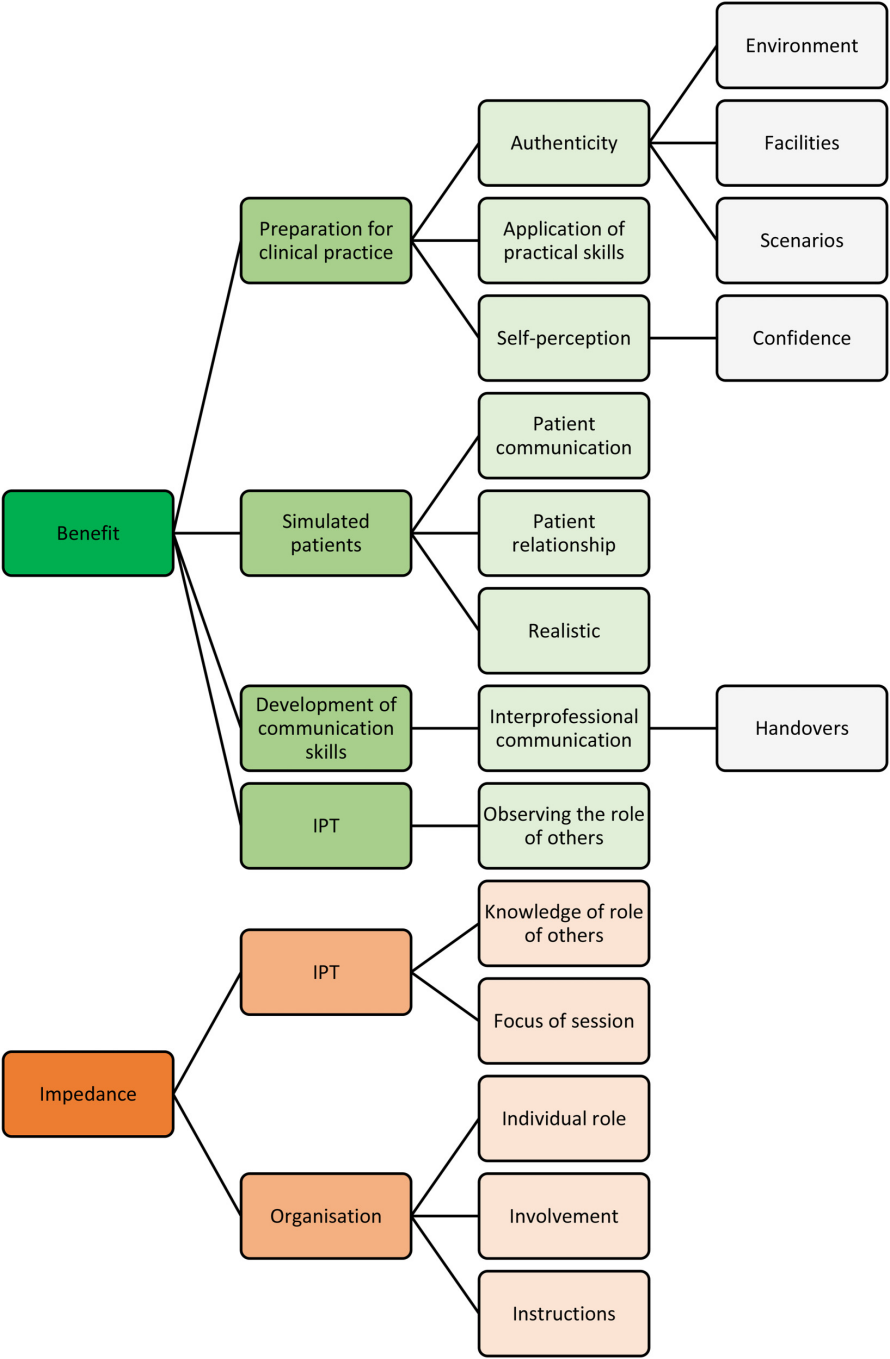
A schematic representation of the iterative thematic process relevant to this study. IPT = interprofessional teamwork.

Qualitative analysis revealed several emergent subthemes and themes which were categorized based on whether they were deemed a benefit or an impediment to learning. This is represented schematically in Figure 3. The results are organized in relation to the session itself, the interprofessional team, and the individual.

**Figure 4.**
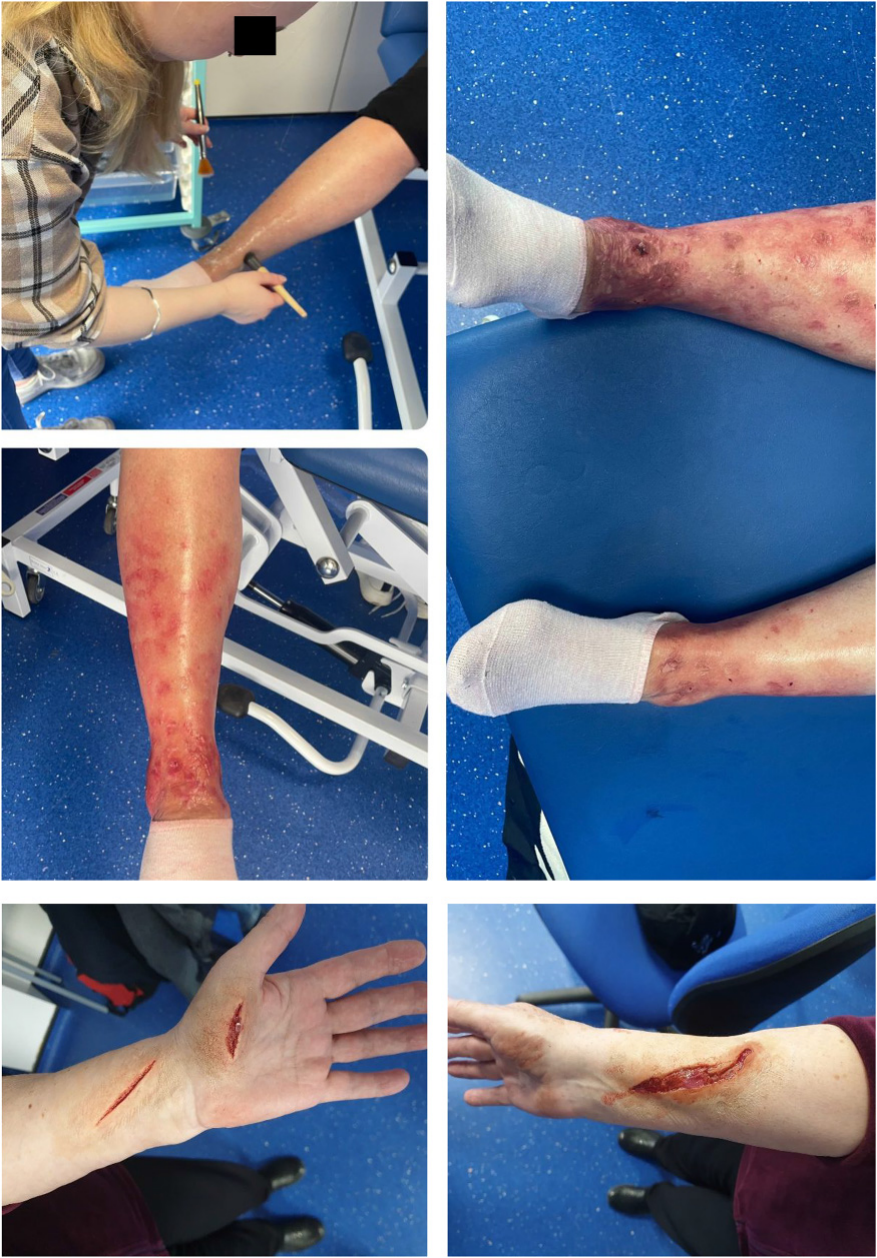
A collation of photographs of special effects from Special Makeup Effects students within the Department of Creative Technologies to promote authenticity.

### The Session

Feedback for the session was largely positive, however, some criticisms were noted. A consistent positive theme was the authenticity of the simulation as supported by student feedback (Figure 4). One student (PM = Paramedic student) believed the environment:

“Gave us an idea of what a hospital ward would be like” (PM 1),

and another felt the session overall gave an:

“insight about real work life” (PA 3).

More specifically, one student liked:

“the way it was laid out with the A&E department and a specialist ward” (PM 10),

while another felt the session helped to:

“get an understanding of how a hospital is ran” (PA 9).

The simulation facilities were only one component of the session contributing to authenticity. Another positive theme which benefited learning was the use of simulated patients. The patient interactions were highly valued across all groups with no negative comments (PHY = Physiotherapy student).

“very good actors, good setting, and realistic” (PA 8).

“she stayed in character really well so it made the situation more realistic” (PHY 1).

Students appreciated being able to interact with the realistic simulated patients and one student found the exposure:

“very beneficial for patient communication” (PA 8).

Through realistic clinical scenarios with professional simulated patients, students described experiencing new clinical experiences, as summarized by one student:

“*the first patient we saw was a cardio-respiratory patient, which was really useful as before this point I hadn't experienced this”* (PHY 1)*.*

Students found the application of practical skills beneficial to their learning with only one commenting on the need for more practical opportunities as a future suggestion. One student reported they:

“enjoyed the change from theory” (PM 4),

and another felt they had:

“gained more practical experience” (PM 6).

The most common critique of the session was the organization. One student felt the session could have been more organized so that:

“the day could have flowed more” and then “everyone could have been more involved” (PM4).

Session involvement was another prevalent theme that emerged as a barrier to learning. As one student noted:

“*There were periods where we weren’t needed throughout the day*…it *would have been nice to have had the chance to see more patients*” (PHY2).

Students also wanted clearer instructions from the organizers. It was reported some of the students were not sure of their tasks and one suggested for:

“a clearer brief so we had more of a job rather than just standing around” (PM1).

Separate from the organizers, support staff present during the simulation received only positive feedback.

### The Interprofessional Team

This session offered a unique opportunity for interprofessional interaction, which received a largely positive response. Specific elements students found beneficial included:

“Working with other professionals” (PM 6).

“to see, watch and learn from others” (PM 9).

Several students agreed the session led to a greater appreciation of the roles of others and one valued the interprofessional approach as:

“a useful learning tool” (PHY 1).

Students specifically valued interprofessional communication and a majority of students felt clinical handovers and communication with colleagues were beneficial to learning. One student valued the team integration aspect of the session for:

“*gaining an understanding of what other professions are responsible for and communicating with them to provide the best possible treatment for the patient*” (PHY 2).

Not all students saw the interprofessional simulation as a positive learning experience. Two students felt they weren’t listened to by other disciplines which impeded learning. One student commented that the session was more focused on one group over the others:

“I feel it was more focused on PA's rather than us as paramedics” (PM 3),

while another student did not value the interprofessional approach:

“I want to be a paramedic not to see if other people can do their job” (PM 2).

Participation within the interprofessional team was another prevalent theme that impeded learning, with paramedic and physiotherapy students reporting they could have been more involved in the session.

“I feel like we could have seen more patients on the ambulance side” (PM 5).

“I’d have liked more physiotherapy cases” (PHY 2).

### The Individual

Several participants reflected on how the session helped their personal development. This included:

“overcoming anxieties” and feeling “more confident by the end of the event” (PM 6).

Elsewhere students enjoyed the challenging environment and one student (PA 5) found taking more responsibility was beneficial to their learning while another felt this (the environment):

“motivated me to study harder” (PA 9).

Students felt the session highlighted their strengths and weaknesses and they were able to reflect on their errors which was beneficial to their own learning. This was summarized by one student's feedback:

“Opportunity…for learning and becoming aware of my strengths and weaknesses” (PHY 2).

“*When reflecting on the day I made notes of things I should look to improve on so when I do simulation again I can perform better”* (PHY 2)*.*

This uncovered another emergent theme of preparedness for clinical practice. Students were positive on the development of key skills including communication, teamwork, and decision-making necessary for their occupation.

“it helped improve my communication and clinical skills” (PA 1).

“It allowed development of my communication skills” (PM 4).

No student reported feeling unsafe or unsupported and only one student reported a negative self-perception and this appeared to be a barrier to their learning:

“felt like I was useless in this situation” (PM 2).

No students reported any psychological or well-being concerns and no students dropped out of the session itself.

## Discussion

To our knowledge, this is the first study of interprofessional simulation involving PA students and other AHPs. By exploring student attitudes and experiences toward interprofessional simulation using a mixed-methods approach, we were able to highlight several benefits and barriers to student learning.

In our study, overall student perception was mainly positive toward the interprofessional simulation session. Students highly valued the authenticity of the session through our realistic clinical environments, simulated patients, and case scenarios; however, student perception was negatively associated with organization and level of involvement within the session. The importance of authenticity has been identified; however, on deeper analysis through focus groups, the authors found the physical environment for simulation was considered less important than organization, interaction, and participation.^33^

Regarding organization, our study did not include any specific briefing or education sessions for students on their own roles or the roles of other professions beforehand. All participants received a briefing on the morning prior to the simulation and for many this could have been their first exposure to working alongside other professions, particularly with PAs, whose roles and competencies are still being established. Clarity of roles can influence engagement^34^ and collaborative practice^35^; however, cross-professional training and role-playing seminars, which can raise awareness of other professionals roles, have been associated with participant dissatisfaction^33^ and higher dropout rates.^15^ Only one negative comment was made regarding the roles of others, whereas the level of guidance and lack of direct instructions were frequent criticisms by the paramedic students, reiterating the importance of experienced instructors and clear instructions.^15,34^

As well as a difference in instructors, the scenarios differed as they were tailored toward separate disciplines, influencing the level of involvement. The importance of participation in authentic teams and within student professional roles has been emphasized^33^; however, it has also been reported when participants felt their involvement or role was insufficient, this impacted their perceived value and was ultimately an obstruction to learning.^36^

While performing in front of students from other professions has the potential for causing anxiety,^37^ there was no evidence in our study to suggest feelings of being judged were barriers to learning. Conversely, students found despite being challenged and making mistakes, they were able to reflect on their own practice and ultimately feel more confident. These findings are consistent with medical and nursing students who recognized emotional strain and stress as positive for enabling learning.^38^

Nevertheless, infrequent but notable comments occurred in the qualitative feedback which suggested reciprocal negative perceptions toward other disciplines. Our study did not specifically measure changes in perceptions, however, in comparison to PAs, paramedic students felt practicing and training with other students less beneficial and were less willing to replace their placement hours with similar simulated learning events in the future. Higher dissatisfaction among paramedic students in a large interprofessional simulation study has been reported and it was suggested differences in level of study and experience were crucial influences.^19^ We did not account for differences in level of study nor previous simulation experience; however, by focusing on the collaborative exchange, rather than individual knowledge, we encouraged interaction on equal levels.^39^ As a result, hierarchical differences within and between professions, which have been reported to impact successful outcomes of simulation training,^39^ were not reported.

**Figure 5.**
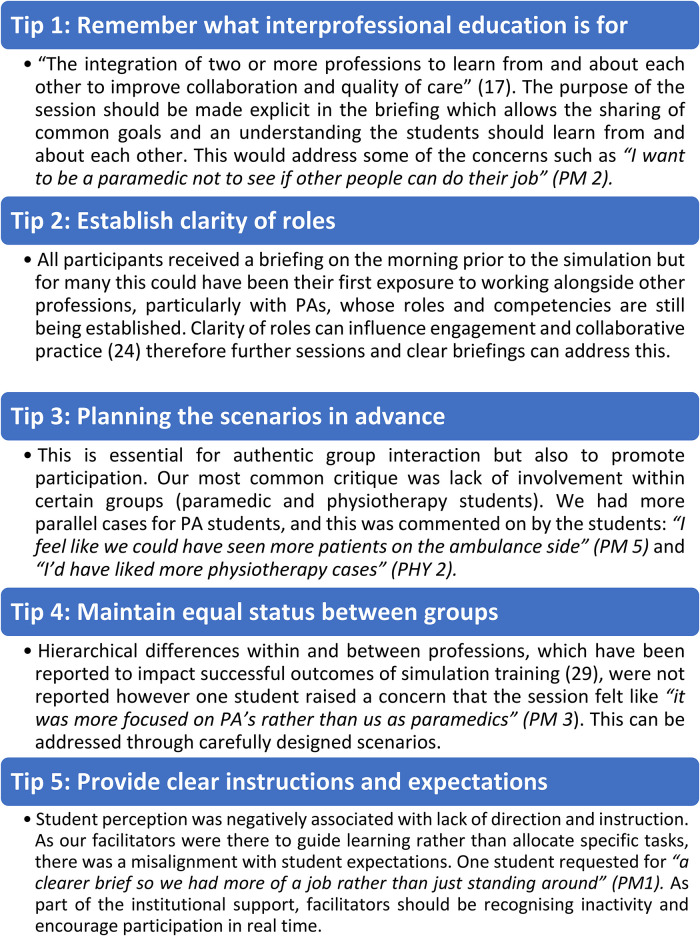
Five top tips to promote effective interprofessional simulation.

The influx of a new healthcare role into a workforce with well-established professions will no doubt pose challenges. At present, it remains unclear which position PAs occupy relative to other AHPs. In our study, at the student level, no group demonstrated authority and collaboration was positively perceived. Interprofessional simulation is an ideal format to provide the necessary conditions for Allport's (intergroup) contact hypothesis to reduce prejudice and improve relationships between groups.^27^ The quality-of-service delivery and patient safety are dependent upon an “effective workforce practising collaboratively” and IPE can aid the development of such a workforce.^20^ Our study contributes to the paucity of literature on interprofessional learning for PAs with the intended wider benefit of improving collaboration. Figure 5 highlights our top tips to promote successful interprofessional simulation as an educational tool.

## Strengths, Limitations, and Future Directions for Research

This is the first study to our knowledge of interprofessional simulation involving PA and AHP students which provides a unique perspective not previously explored. The use of HFS with expert-designed clinical scenarios promoted authenticity and self-perceived preparation for clinical practice.

Despite demonstrating interprofessional simulation is well accepted by our student participants, several limitations exist. Firstly, this is a single-center study and is not easily reproducible due to the unique facilities we have available. Secondly, we did not perform a sample size or power analysis before conducting the study and as it was voluntary, we had a small sample size which limited statistical analysis to compare differences between courses.

Although there were no existing questionnaires authenticated for PA students and our survey was based on well-established scales for IPE, it was not validated. Similarly, we did not use a pre and posttest questionnaire and so were unable to measure changes in perception, although this was not the primary aim of this pilot study. Additionally, we relied on student self-perception of confidence and satisfaction which only reaches level 2 of Kirkpatrick's hierarchy and therefore we cannot comment on the degree of learning or change to clinical practice.^40^ Furthermore, we did not adopt specific indirect measures of anxiety or stress levels to corroborate student self-perception; therefore, our conclusions regarding this are speculative.

In future, we intend to repeat our simulation on a larger scale. We plan to utilize validated pre- and post-session evaluation tools. Further research has been approved to evaluate subsequent sessions using the Utilization-Focused Evaluation framework.^41^ The initiative is a low level of evaluand maturity suggesting developmental evaluation is better suited and to achieve proper alignment we aim to include focus groups for students and staff to engage key stakeholders.

## Conclusion

To our knowledge, this is the first study of interprofessional simulation involving PA students and other AHPs. By designing and implementing this initiative, interprofessional simulation has now been incorporated into the syllabus for the PA, paramedic, and physiotherapy courses. This is in keeping with HEE’s plan for the development of a well-trained multiprofessional workforce through simulation-based education.

By exploring student attitudes and experiences toward interprofessional simulation using a mixed-methods approach, we were able to highlight several benefits and barriers to student learning which were shared at a national PA conference with other Higher Education institutions.

Our long-term aim is to use high-fidelity interprofessional simulation to assess behavioral changes, but further studies would be necessary to determine the impact in real clinical practice.

## Supplemental Material

sj-docx-1-mde-10.1177_23821205241249594 - Supplemental material for An Exploration of Student Perception Toward Interprofessional High-Fidelity Clinical Simulation Supplemental material, sj-docx-1-mde-10.1177_23821205241249594 for An Exploration of Student Perception Toward Interprofessional High-Fidelity Clinical Simulation by Michael Hannides, Rameez Tariq, Mark Holland, Polykarpos Angelos Nomikos, Rory McKelvin and Michelle Powell in Journal of Medical Education and Curricular Development

sj-docx-2-mde-10.1177_23821205241249594 - Supplemental material for An Exploration of Student Perception Toward Interprofessional High-Fidelity Clinical Simulation Supplemental material, sj-docx-2-mde-10.1177_23821205241249594 for An Exploration of Student Perception Toward Interprofessional High-Fidelity Clinical Simulation by Michael Hannides, Rameez Tariq, Mark Holland, Polykarpos Angelos Nomikos, Rory McKelvin and Michelle Powell in Journal of Medical Education and Curricular Development
