# Supplementary figures and images for: An Exploration of Student Perception Toward Interprofessional High-Fidelity Clinical Simulation
Source: J Med Educ Curric Dev. 2024 Apr 25;11:23821205241249594. doi: 10.1177/23821205241249594 (PMC11044798; doi:10.1177/23821205241249594)

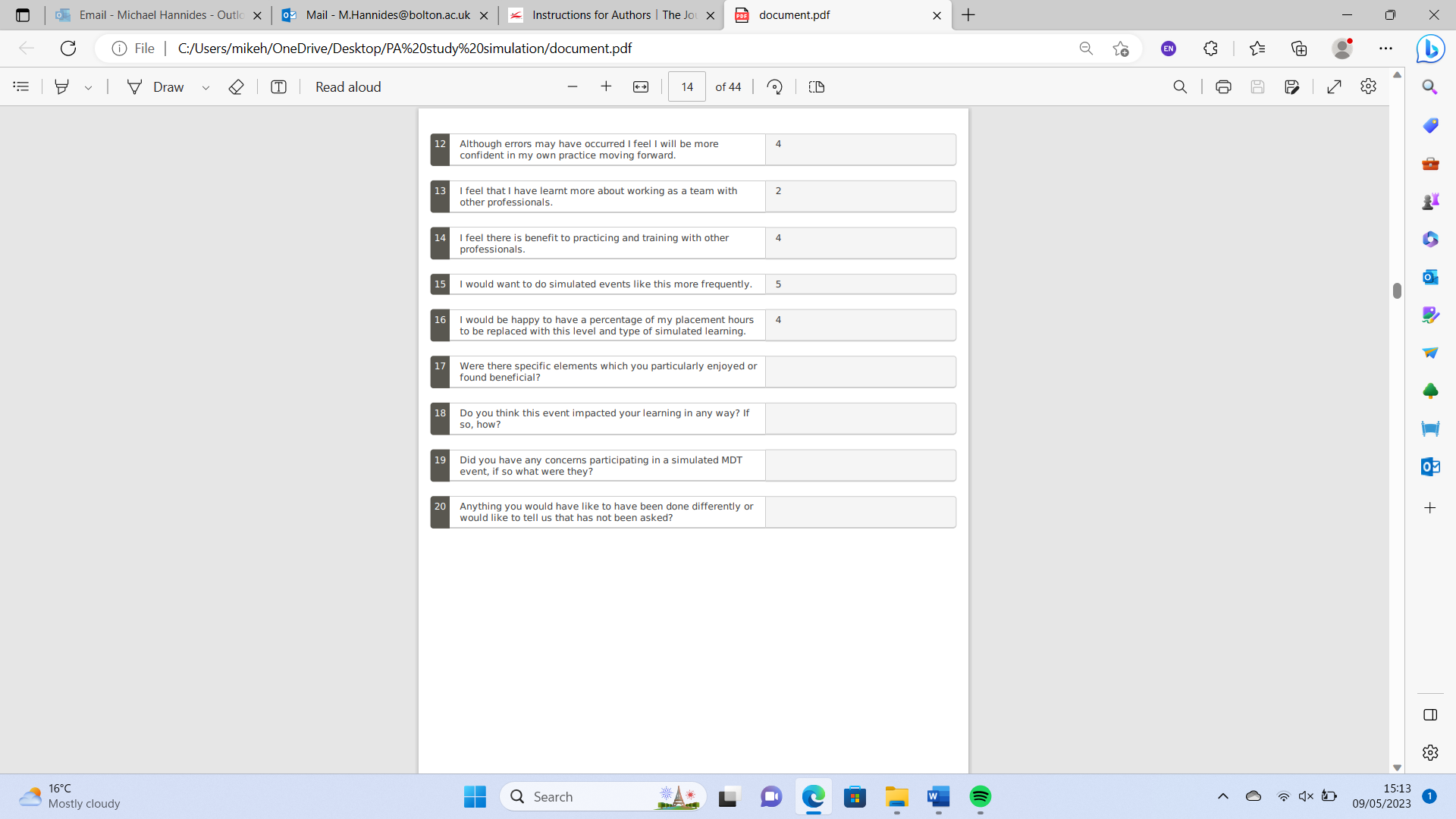

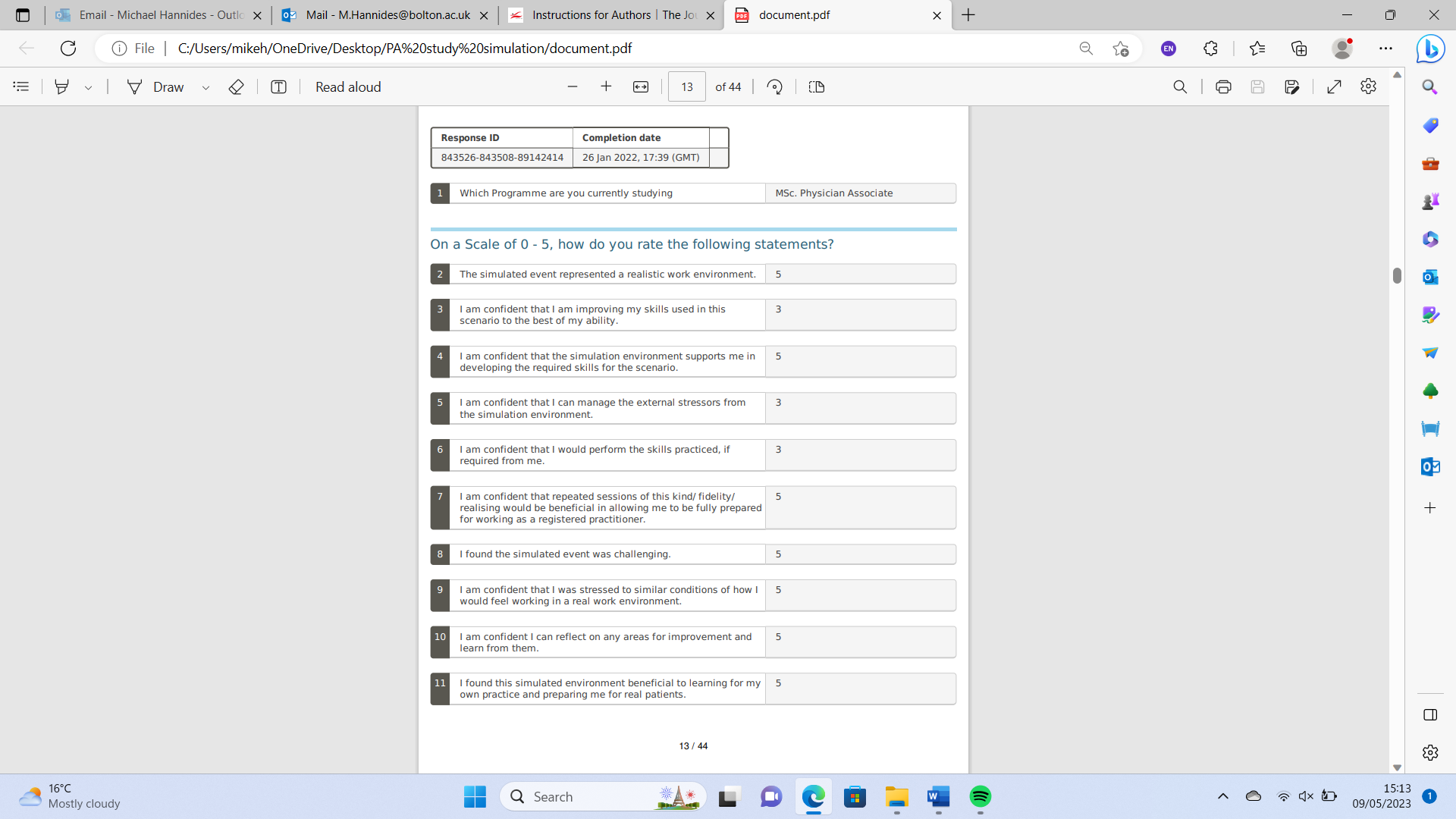

Supplement: sj-docx-2-mde-10.1177_23821205241249594 - Supplemental material for An Exploration of Student Perception Toward Interprofessional High-Fidelity Clinical Simulation [file sj-docx-2-mde-10.1177_23821205241249594.docx]
